# Care technologies to prevent and control hemorrhage in the third stage of labor: a systematic review*

**DOI:** 10.1590/1518-8345.2761.3165

**Published:** 2019-08-19

**Authors:** Rita de Cássia Teixeira Rangel, Maria de Lourdes de Souza, Cheila Maria Lins Bentes, Anna Carolina Raduenz Huf de Souza, Maria Neto da Cruz Leitão, Fiona Ann Lynn

**Affiliations:** 1 Universidade Federal de Santa Catarina, Florianópolis, SC, Brasil.; 2 Universidade do Vale do Itajaí, Itajaí, SC, Brasil.; 3 Universidade do Estado do Amazonas, Manaus, AM, Brasil.; 4 Prefeitura Municipal de Florianópolis, Secretaria Municipal de Saúde, Florianópolis, SC, Brasil.; 5 Escola Superior de Enfermagem de Coimbra, Coimbra, Portugal.; 6 Queens University, School of Nursing, Belfast, Irlanda del Norte

**Keywords:** Postpartum Hemorrhage, Biomedical Technology, Disease Prevention, Diffusion of Innovation, Maternal Death, Nursing Care, Hemorragia Pós-Parto, Tecnologia Biomédica, Prevenção de Doenças, Difusão de Inovações, Morte Materna, Cuidados de Enfermagem, Hemorragia Posparto, Tecnología Biomédica, Prevención de Enfermedades, Difusión de Innovaciones, Muerte Materna, Atención de Enfermería

## Abstract

**Objective:**

to identify evidence concerning the contribution of health technologies used to prevent and control hemorrhaging in the third stage of labor.

**Method:**

systematic review with database searches. First, two researchers independently selected the papers and, at a second point in time, held a reconciliation meeting. The Kappa coefficient was used to assess agreement, while the Grading of Recommendations, Assessment, Development and Evaluation was adopted to assess risk of bias and classify level of evidence.

**Results:**

in this review, 42 papers were included, 34 of which addressed product technologies, most referred to pharmacological products, while two papers addressed the use of blood transparent plastic bags collector and the contribution of birth spacing and prenatal care. The eight papers addressing process technologies included the active management of the third stage of labor, controlled cord traction, uterine massage, and educational interventions.

**Conclusion:**

product and process technologies presented high and moderate evidence confirmed in 61.90% of the papers. The levels of evidence confirm the contribution of technologies to prevent and control hemorrhaging. Clinical nurses should provide scientific-based care and develop protocols addressing nursing care actions.

## Abstract (pt)

**Objetivo:**

identificar evidências acerca das contribuições das tecnologias de cuidado usadas para prevenção e controle da hemorragia no terceiro estágio do parto.

**Método:**

revisão sistemática com busca em bases de dados. Dois investigadores selecionaram os textos de forma independente na primeira etapa e, na segunda, em reunião de conciliação. Para avaliação da concordância, aplicou-se o coeficiente Kappa; para avaliação do risco de viés e classificação dos níveis de evidência, adotou-se o *Grading of Recommendations, Assessment, Development and Evaluation*.

**Resultados:**

incluíram-se 42 artigos; desses, 34 classificados como tecnologias de produto, sendo a maioria produtos farmacológicos; dois referentes ao uso do saco plástico transparente para a coleta de sangue e contribuição do intervalo de nascimento e dos cuidados pré-natais. Os oito artigos classificados como tecnologias de processo se referiam a manejo ativo do terceiro estágio do parto, tração controlada de cordão, massagem uterina e intervenções educacionais.

**Conclusão:**

as tecnologias de produto e de processo apresentaram evidência alta e moderada confirmada em 61,90% dos artigos. Os níveis de evidência demonstram contribuições das tecnologias para prevenção e controle da hemorragia. Na prática clínica, o enfermeiro deve oferecer cuidados à mulher fundamentados em evidências científicas e construir protocolos sobre as ações de cuidado da enfermagem.

## Abstract (es)

**Objetivo:**

identificar las evidencias acerca de las contribuciones de las tecnologías de cuidado usadas para la prevención y el control de la hemorragia en la tercera etapa del parto.

**Método:**

revisión sistemática con búsqueda en bases de datos. Dos investigadores seleccionaron los textos, de forma independiente, en la primera etapa; y, en la segunda en reunión de conciliación. Para evaluación de la concordancia fue aplicado el coeficiente Kappa; para evaluación del riesgo de sesgo y clasificación de los niveles de evidencia, se adoptó el *Grading of Recommendations, Assessment, Development and Evaluation*.

**Resultados:**

fueron incluidos 42 artículos; de estos, 34 fueron clasificados como: tecnologías de producto (siendo la mayoría productos farmacológicos), dos referentes a la contribución del saco plástico transparente recolector de sangre y del intervalo de nacimiento y de los cuidados prenatales. Los ocho artículos clasificados como tecnologías de proceso se referían al manejo activo de la tercera etapa del parto, a la tracción controlada del cordón, al masaje uterino y a intervenciones educacionales.

**Conclusión:**

las tecnologías de producto y de proceso presentaron evidencia alta y moderada, lo que fue confirmado en 61,90% de los artículos. Los niveles de evidencia demuestran las contribuciones de las tecnologías para la prevención y el control de la hemorragia. En la práctica clínica, el enfermero debe ofrecer cuidados a la mujer fundamentados en evidencias científicas y construir protocolos sobre las acciones de cuidado de la enfermería.

## Introduction

Postpartum hemorrhage (PPH) is one of the main causes of maternal morbidity and mortality worldwide^1-2^. PPH is defined as blood loss above 500 ml, measured up to 24 hours postpartum, while this amount of blood loss after 24 hours is defined as secondary PPH^1,3^. Blood loss up to 500ml among healthy women does not lead to negative consequences; however, uncontrolled blood loss over 500ml can be fatal^1^. Primary PPH occurs in the first 24 hours after birth and is more likely to result in maternal morbidity and mortality, while secondary postpartum hemorrhage refers to bleeding that occurs from 24 hours up to six weeks after birth^1,3^.

In general, blood loss is diagnosed as PPH if one or more of the following occur: loss of uterine tone (atony); retention of placental tissue or blood clots; laceration of the genital tract; or coagulopathy^1,3^. Procedures to prevent PPH are initiated by assessing a patient’s risk profile and establishing how to respond to complications in order to prevent a small amount of bleeding from becoming a severe hemorrhage with the risk of death. PPH is one of the complications of the third stage of labor and this stage begins after the fetus is expelled; however, with the detaching of the placenta from the uterine wall and its expulsion through the birth canal, greater than expected bleeding may occur. Therefore, it is essential to know the physiology of childbirth and women’s clinical conditions, as well as intercurrences that took place during the pregnancy-puerperal period, which might contribute to the emergence of hemorrhaging^1,4^.

In order to prevent PPH, the staff needs to be prepared to use protocols with a multidisciplinary approach, which involves maintaining hemodynamic stability while, simultaneously, identifying and treating the cause of bleeding. A combination of prediction and prevention, early identification and rapid coordinated actions is essential to preventing PPH. Consequently, efficient communication among the members of the multidisciplinary obstetrical team is paramount^5^.

Prevention and control of PPH demand technologies that support labor and interventions in the event unwanted bleeding occurs. Therefore, health workers should be aware of and implement technologies supported by a higher level of evidence and with positive outcomes, which represent the least harm to women and babies. Additionally, for safe and timely care to be provided, services need to have a well-established capacity to coordinate people, equipment and work processes. Hence, having techniques and technologies as well as protocols does not ensure, by itself, the prevention and control of hemorrhaging; personnel of sufficient quality and number to meet demand is necessary.

Technologies, evidence-based practice, and interventions proposed by workers have grown exponentially in importance from the mid-twentieth century, so much so that providing quality services without such resources is inconceivable currently, with many of them being innovative in nature^6^. All these aspects a service is required to have are known as health technologies – a term that encompasses every intervention used to promote health. “In this sense, health technologies can be conceived as the practical application of knowledge, including machines, clinical and surgical procedures, medications, programs and systems intended to promote health care”^7^.

Similarly, the literature presents the elements that integrate health technologies, namely any and all methods/devices used to promote health, prevent death, and treat diseases and improve rehabilitation or the care of individuals or populations^8^.

Traditional product technologies (equipment, drugs and material) require intermediate steps with relatively differentiated processes and actors, although these are increasingly connected. Note that product technologies such as diagnostic or therapeutic resources used in healthcare delivery are more effective when combined with process technologies. Process technologies, in turn, include operational procedures, care, education and management techniques. Thus, one should seek a scientific basis to strengthen healthcare delivery and produce evidence to innovate in regard to product and process technologies^9-10^.

There are various systematic reviews addressing PPH showing it still accounts for high levels of morbidity and mortality, mainly in developing countries. For this reason, studies need to be conducted regularly, considering that all technologies should be revised and updated over time, especially when we consider the application of technologies in different contexts and populations^6-8,11^.

In order to overcome the causes of PPH and its determinants of a sociocultural, technical and technological nature in different geo-economic contexts, the results of systematic reviews should be disseminated among health services in order to contribute to the development of multidisciplinary protocols. In this context, nurses, especially those in the obstetrical field, can propose care processes, among others, such as those associated with patient safety, especially when applying pharmacological products^11^.

Given the importance of hemorrhaging, which may be associated with diverse events such as uterine atony, uterine rupture, blood dyscrasia, cephalopelvic disproportion and placental abruption^1^, the need to prepare the staff to intervene appropriately, and lack of information regarding existing technologies in the field, the objective of this study was to identify evidence concerning the contribution of health technologies used to prevent and control hemorrhaging in the third stage of labor.

## Method

This Systematic Review adopted the PRISMA (Preferred Reporting Items for Systematic Reviews and Meta-Analyses) checklist^12^ to present the results. The entire review process was guided by the question: What is the evidence available concerning the contributions of health technology used to prevent and control hemorrhaging in the third stage of labor?” The PIO (Patient, Intervention and Outcomes) support protocol^13^ was used, in which P (population, participants) was represented by women with blood loss in the third stage of labor; I (intervention/procedure) was represented by technologies used to prevent PPH; and O (outcome) was the occurrence of PPH, level of blood loss, or success in managing the decrease of harm to women.

Data were collected from July 10 to 12, 2016 from the following databases: Latin American and Caribbean Health Sciences (LILACS), Database of Nursing (BDENF), Scientific Electronic Library Online – Brazil (SciELO - Brazil), National Library of Medicine (PubMed)/Medical Literature Analysis and Retrieval System on Line (MEDLINE) and Scopus. The period between June 2006 and July 2016 was chosen because we deemed it would contain the most current technologies. Studies, the title or abstract of which addressed the topic and were available in Portuguese, Spanish or English, were identified from July 13^th^ to December 30^th^ 2016. The search strategy included the following descriptors postpartumhemorrhage; hemorrhage; postpartumperiod; labor stage, third, which were adapted for MeSHTerms All Fields - hemorrhage; hemorrhages; hemorrhagic; bleeding; postpartum; puerperal; Third Stage. DeCS terms – postpartumhemorrhage [postpartumhemorrhage]; *Hemorragia Pós-Parto* [Postpartum Hemorrhage]*; Hemorragia Puerperal* [Puerperal Hemorrhage]*; Hemorragia Posparto* [Postpartum Hemorrhage]*; hemorragia* [hemorrhage]*; sangramento* [bleeding]*; Período Pós-Parto* [Post-partum Period]*; pos-parto* [post-partum]*; pós-parto* [post-partum]*; Terceira Fase do Trabalho de Parto* [Third Stage of Labor]. The following key words were used: *Terceiro período* [Third period]*; Terceiro estágio* [Third stage]*; Terceira etapa* [Third stage]. The Boolean expressions “AND”, “NOT” and “OR” were used to locate the instances the aforementioned descriptors occurred simultaneously.

Two independent researchers selected the papers, examining each paper’s title, abstract and full text according to the following inclusion criteria: studies addressing technologies to prevent hemorrhaging in the third stage of labor; home delivery or hospital delivery; delivery assisted by any birth assistance or traditional birth assistance; randomized clinical trial (RCT) or quasi-randomized, observational studies, or analytic descriptive studies. Exclusion criteria were: theses, dissertations, editorials, integrative or systematic reviews, descriptive observational studies, and qualitative studies.

After selecting the papers, the researchers held a meeting to reconcile agreements and disagreements by carefully consulting the full texts. Of the 48 papers selected, the researchers disagreed in regard to 10. After discussion, they decided to include four of these papers, with 42 papers being included in the final review. The Kappa coefficient^14-18^, with a confidence interval of 95%, was applied to assess inter-rater agreement. This coefficient has the following measure levels: less than zero, “insignificant”; between 0 and 0.2, “weak”; between 0.21 and 0.40, “reasonable”; between 0.41 and 0.60, “moderate”; between 0.61 and 0.80, “strong”; and between 0.81 and 1.0, “almost perfect”.

**Figure 1:**
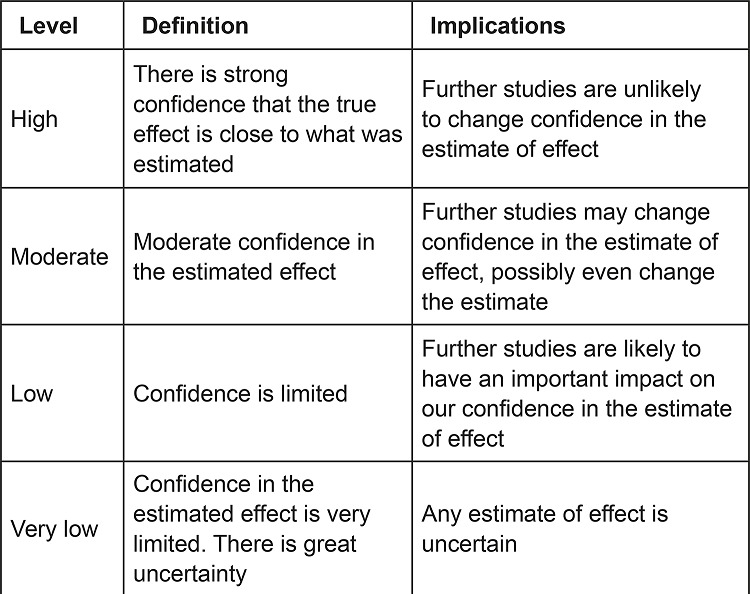
– Evidence levels. Florianópolis, SC, Brazil, 2018

Source: Adapted by the authors^(19)^.

The levels of evidence identified in the papers were classified according to Grading of Recommendations Assessment, Development and Evaluation (GRADE)^19-20^, a system sensitive to rating the quality of evidence. In this system, quality of evidence is classified as: high, moderate, low, or very low (Figure 1). Evidence originating from randomized clinical trials starts with a high level and evidence from observational studies with a low level.

In this study, the classification used (the GRADE system) to assess the quality of evidence took into account the risk of bias in randomized clinical trials addressing product technologies, in terms of methodological limitations concerning the design or implementation of individual studies. Evidence of randomized clinical trials may be downgraded if allocation is not confidential; absence of blinding; incomplete follow-up; a report of selective outcomes, and other limitations, such as: early interruption of the study due to some benefit or insufficient information to assess whether there is important risk of bias. Risk of bias was assessed for each of these domains and classified as high-risk, uncertain, or low risk of bias^20^.

In the third stage of the process, after reading the full texts and excluding those that failed to meet the inclusion criteria, according to two independent researchers, we proceeded to the systematization of studies.

In order to compile and synthetize the results of the different studies included in the review, we organized tables and grouped the technologies into two categories: product technologies and process technologies. Tables are presented in the results.

## Results

After applying the search strategies, 6,999 papers were found. Of these, 6,726 were excluded due to the following: the titles and/or abstracts of 5,978 papers did not meet the inclusion criteria; 652 papers were published in more than one database; and 96 were not characterized as papers (e.g., theses, dissertations, integrative literature reviews, descriptive observational studies, papers other than scientific research, and qualitative studies)

**Figure 2:**
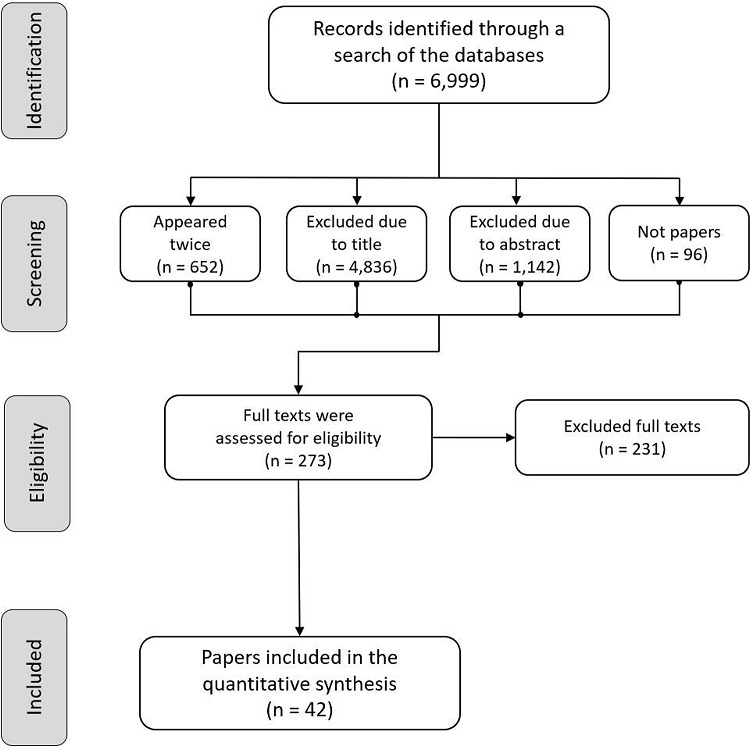
Flowchart PRISMA concerning the studies’ identification and screening process. Florianópolis, SC, Brazil, 2018

The full texts of 273 papers were read and 42 were included (Figure 2). The Kappa coefficient^14-18^, which was equal to 0.86 in the first stage and equal to 1.00 in the second, revealed a high level of inter-rater agreement.

**Figure 3:**
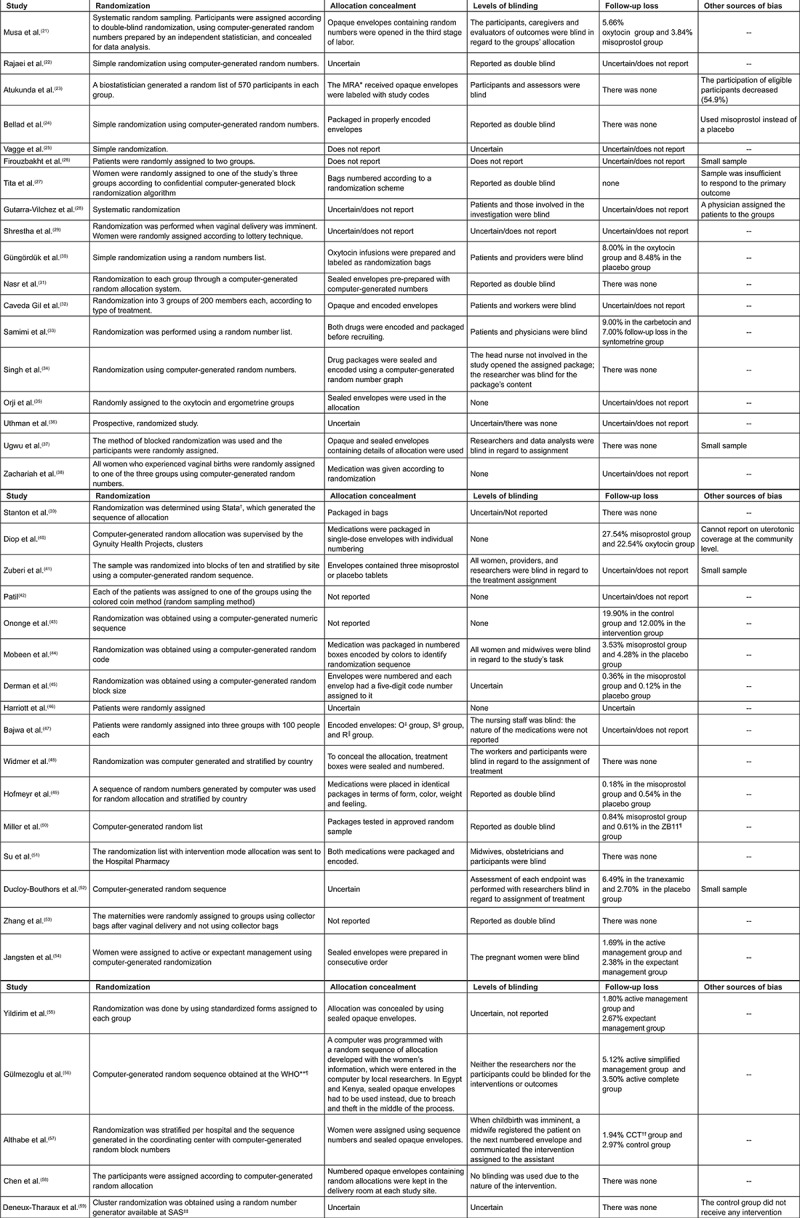
*MRA - Midwife Research Assistants; †Stata - Statistical Software for Data Science, ‡O - Oral; §S - Sublingual; ║R - Rectal; ¶ZB11 - Zhi Byed 11; **WHO – World Health Organization; ††CCT – Controlled Cord Traction; ‡‡SAS – Statistical Analysis Software. – Risk of bias in randomized clinical trials classified as technologies to prevent and control hemorrhage in the third stage of labor. Florianópolis, SC, Brazil, 2018

**Figure 4:**
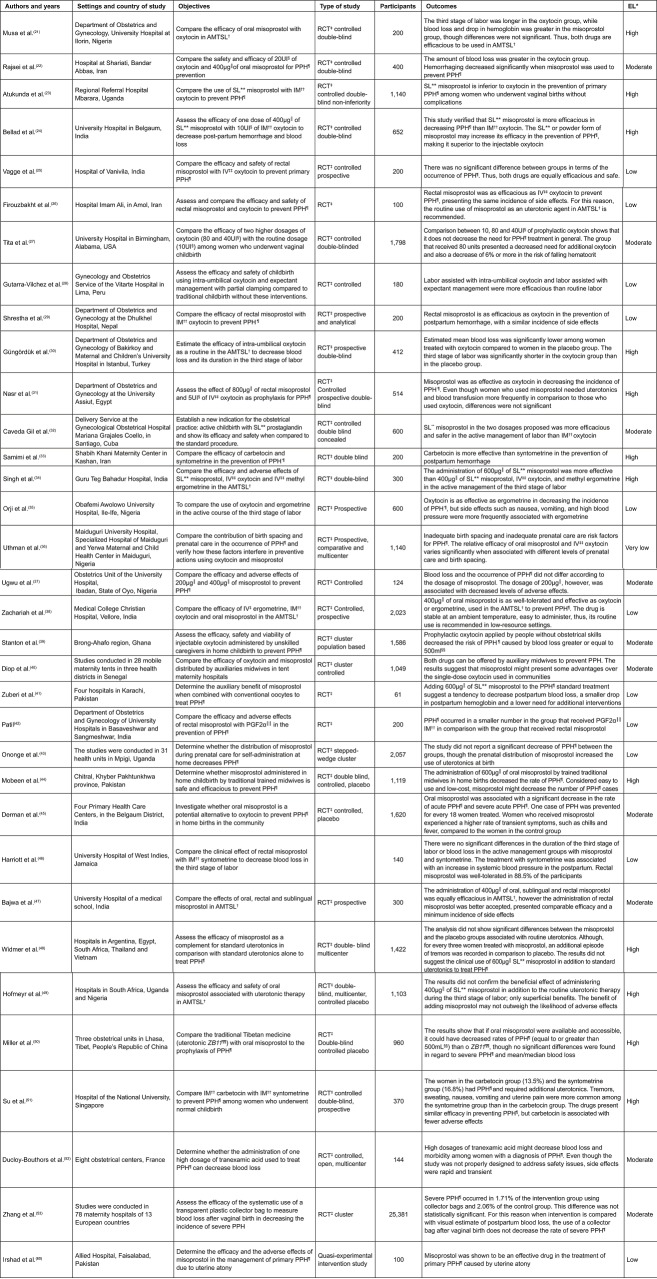
*EL – Evidence Level; †AMTSL - Active Management of the Third Stage of Labor; ‡RCT – Randomized Clinical Trial; §IU – International Units; ║µg - Microgram; ¶PPH - Postpartum Hemorrhage; **SL - Sublingual; ††IM - Intramuscular; ‡‡IV - Intravenous; §§mL - Milliliter; ║║PGF2α - Prostaglandin F2α; ¶¶ZB11 *- ZhiByed 11*. – Distribution of papers classified as product technologies. Florianópolis, SC, Brazil, 2018

**Figure 5:**
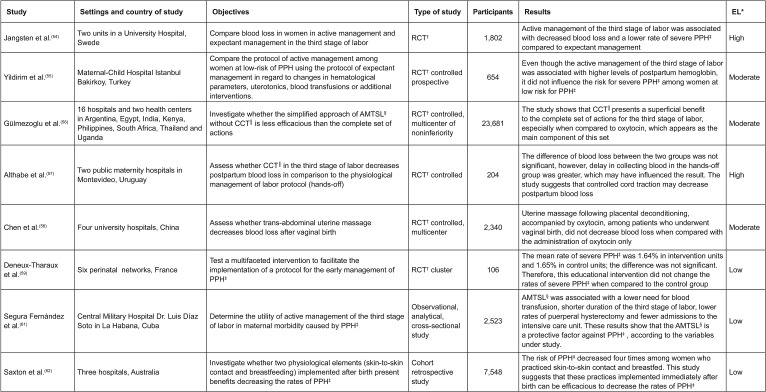
*EL – Evidence Level; †RCT – Randomized Clinical Trial; ‡PPH – Postpartum Hemorrhage; §AMTSL - Active Management of the Third Stage of Labor; ║CCT – Controlled Cord Traction. – Distribution of papers classified as process technologies. Florianópolis, SC, Brazil, 2018

Regarding the methods used by the papers under study, 39 are RCTs; one is a quasi-experimental study; one is a retrospective cohort; and one is an observational study. The risk of bias was assessed in the 39 randomized clinical trials addressing technologies to prevent and control hemorrhaging in the third stage of labor using the GRADE system (Figure 3). The findings show that 14 papers present High Level evidence (35.9%), 12 present Moderate Level evidence (30.8%), and 13 present Low Level evidence (33.3%). The remaining studies (quasi-experimental, retrospective cohort and observational studies) maintained the original Low Level evidence (Figures 4 and 5).

The papers were classified as product and process technologies^9^. Among the 34 papers classified as product technologies (Figure 4), most were represented by pharmacological products and only two papers refer to another type of product, that is, plastic collector bag and the contribution of birth spacing and prenatal care.

Thirty, out of the 34 papers classified as product technologies addressed technologies directed to the prevention of PPH, including eight papers that accessed the isolated use of misoprostol; four addressed the use of oxytocin through different routes and dosages; 12 studies compared misoprostol to oxytocin; two studies compared carbetocin to syntometrine; one analyzed ergometrine *versus* oxytocin; one ergometrine *versus* oxytocin *versus* misoprostol; one addressed PGF2α (Prostaglandin F2α) *versus* misoprostol; and one compared ZB11 *(ZhiByed 11 –* Tibetan traditional medicine) to misoprostol. Product technologies to prevent PPH encompassed studies that presented interventions using uterotonic drugs and transparent plastic collector bag.

Eight papers were classified as process technologies (Figure 5), which included: active management of the third stage of labor; controlled cord traction; skin-to-skin contact and breastfeeding; sustained trans-abdominal uterine massage; and educational intervention.

## Discussion

The evidence supports decision-making concerning clinical practice and gives rise to new ways to approach a problem over time. Changes in care practice, however, are slow considering that the production of knowledge and its incorporation require qualified personnel, as the application of evidence in care practice is a responsibility of not only one profession but of the entire care system; thus, it is a responsibility of all the professionals in institutions and of society itself. Therefore, one has to consider the sociocultural and educational context, as well as safety culture and the innovation taking place in health services concerning practice, intervention and outcomes. Otherwise, evidence may become innocuous if professionals are mobilized by repetition rather than innovation, commitment and responsibility to life.

It is, therefore, based on this assumption that we present evidence reported by the 42 studies that make up this systematic review: 33 of these analyzed the efficacy of uterotonic drugs to prevent/treat PPH, including studies addressing oxytocin, misoprostol, ergometrine, syntometrine, carbetocin, PGF2α and ZB11. Oxytocin and misoprostol appear as the most studied drugs, with results for them being presented in a larger number of publications.

In regard to the product technologies, most papers addressed the use of uterotonics during the third period of labor and recommend them to prevent PPH. The studies addressing the efficacy of different dosages of synthetic oxytocin, report a shorter duration of the third stage of labor^21-31^, when compared to other drugs, regardless of the dosage and route of administration, with the exception of one study^32^.

In regard to blood loss, six studies^22,24,32-35^ report that oxytocin presented a mean blood loss. Birth spacing influences this result; that is, greater blood loss in found among childbirths with intervals of less than two years^36^. Other studies^21,25-26,28,30^ did not report significant differences in regard to this aspect.

Significant differences were not reported by the studies addressing oxytocin in the third stage of labor in regard to the occurrence of PHH and/or severe PPH and drop in the concentration of hematocrit/hemoglobin^21-23,25-27,31,37-38^. Other studies^22,24^, however, presented a greater occurrence of PPH in the oxytocin group, requiring a greater use of additional uterotonics in this group. The employment of oxytocin in intervals shorter than two years between births determined a greater occurrence of PPH^36^. Additionally, a drop in the concentration of hematocrit in the oxytocin group was significant in the postpartum^24,32^.

The administration of oxytocin, even by unskilled individuals, was efficacious to control PPH and also to decrease blood loss, as well as the need to refer patients to referral units^39^. One study^40^, however, reports greater amounts of blood loss in the oxytocin group.

The use of misoprostol was analyzed in studies with different compositions and administration routes to prevent PPH^21-26,29,31-32,37,41^. These studies show that, regardless of the dosage of misoprostol, the duration of the third stage of the labor did not present significant differences, nor did the drop in the concentration of hematocrit/hemoglobin, except in those papers^24,32^ that found a decrease in the concentration of these hematological components.

Studies report that both that the duration of the third period of labor and mean loss of blood were significantly longer and greater^23,29,42-43^ and shorter and lower^22,24,32,34,37,41^.The occurrence of PPH and/or severe PPH was greater among women who received misoprostol^22,43-44^. The RCTs^21,26,32,36,46^ report that misoprostol was more efficacious in preventing PPH among women with inadequate birth spacing, that is, less than two years. This drug/dosage/route was effective when used in the treatment of primary PPH caused by uterine atony^59^.

When considering the route of administration, sublingual misoprostol was more efficacious, decreasing the duration of the third stage of labor and mean blood loss in comparison to the rectal and oral routes. Conversely, side effects like fever and tremors were more frequent when the sublingual route was used. The group who received the medication through the oral route presented a greater need for additional uterotonics. In terms of acceptability, rectal misoprostol was better accepted by women and was the one with fewer side effects^46-47^.

Side effects were common in all women who received misoprostol. Most studies comparing oxytocin to misoprostol report significant differences in which groups treated with misoprostol experienced more side effects, especially tremors and fever^21-26,29,31,34,37-38,42,44-45,47-50^, with the exception of one study^32^ that did not report significant differences. Other symptoms like nausea, vomiting and diarrhea were found, but no significant differences were found between groups^21-26,29,31,38,43,50^.

Seven studies^21,23,25,31,43-45^ recommend misoprostol be adopted in areas where routine uterotonics are not available, as it is the best option to prevent PPH, given its ease of application, stability at ambient temperature and low cost. This is a safe and efficacious alternative to be used by midwives and auxiliaries in home births. Misoprostol is the most viable choice in communities^40^ and is easy to store^42^. In areas where there are routine uterotonics available, however, the benefits of misoprostol might not outweigh the discomfort of side effects^49^.

In regard to the viability of using misoprostol in communities, a study that distributed it during prenatal care for women to self-administer it at home, no significant decrease of PPH was found. Nonetheless, the use of uterotonics increased as did the return visits of women to the health service. The self-administration of misoprostol with little monitoring and supervision was considered safe^43^, though women need to be better educated on when to use misoprostol in regard to placental deconditioning.

In regard to misoprostol, RCTs present a high level of evidence^21,24,31,34,40,44,50^ recommending the use of this uterotonic via the oral, sublingual and rectal routes in different dosages, indicating it is efficacious, economically viable and easy to administer. Therefore, the use of misoprostol in areas with few resources is a good alternative to oxytocin. Analysis of bias indicated there were no methodological limitations regarding the design or implementation of the individual papers presented. The RCTs^43,59^ with moderate evidence indicate that oral misoprostol is the only pharmacological option available in areas with few resources to prevent postpartum hemorrhaging and bleeding. Other studies^25-26,29,41-43,46^ addressed in this review and that address the use of misoprostol also make similar recommendations but, in accordance with GRADE (Figure 3), present methodological limitations. Further studies need to focus on the potential efficacy of misoprostol in areas where standard uterotonics are not available.

In regard to the use of oxytocin, RCTs with moderate level of evidence^35,39^recommend the prophylactic use of misoprostol to prevent PPH, while this is an essential intervention. Thus, oxytocin is the drug of choice whenever available and with a 10UI dosage; it is as efficacious as ergometrine in decreasing the incidence of PPH, though without the undesirable side effects associated with ergometrine. These studies present methodological limitations regarding the blinding of participants.

As noted in the previous paragraph, in addition to misoprostol and oxytocin, the efficacy of ergometrine, syntometrine and PGF2α in preventing PPH was also compared. Studies addressing ergometrine^33-35,38^report no significant differences in regard to the remaining uterotonics assessed in terms of blood loss, drop in hematocrit, duration of the third stage of labor, or the additional need of other drugs. The risk of side effects (nausea, high blood pressure, headaches and vomiting), however, was greater in the group receiving ergometrine. For this reason, the recommendation to use ergometrine depends on the relevance of risks^35^.

In regard to syntometrine, the studies intending to determine the severity and incidence of this drug’s side effects report no significant differences in terms of the duration of the third stage of labor, amount of blood loss, and use of additional uterotonics^33,46^. Significant differences, however, were reported by another study^51^, in regard to nausea, tremors, vomiting, uterine pain, and sweating.

In the comparison performed between syntometrine and carbetocin in the prevention of PPH^33,51^, carbetocin was more efficacious than syntometrine, though one of the studies^51^ does not report relevant differences between the efficacies of both. The study with carbetocin identified fewer side effects and, even though an analysis of cost/efficacy was not performed, the author reports that the cost of carbetocin is ten times greater than the cost of syntometrine, while emphasizing that carbetocin was associated with fewer side effects, so that its use can contribute to reduced costs and time required for professionals.

PGF2α in one RCT^42^ was more efficacious than misoprostol in decreasing mean blood loss and the duration of the third stage of labor and the drop in hemoglobin levels. The associated gastrointestinal side effects, however, were significantly greater and included nausea, vomiting, diarrhea and abdominal cramps.

One RCT^50^ conducted in China addressed the ZB11 uterotonic, used in Tibetan traditional medicine to prevent PPH. The results show higher rates of PPH in the ZB11 group in comparison to the misoprostol group. No significant differences were found in terms of blood loss. Side effects such as diarrhea, tremors, and fever were less recurrent. The authors^50^ suggest that other studies be undertaken in that geographic area because home births performed by the pregnant women themselves, or without the assistance of qualified workers, is not uncommon. Thus, research addressing efficacious uterotonics with accessible prices is especially relevant in areas with these characteristics and can contribute to women’s easier access to safe technologies^50^. This specific RCT was assessed using the GRADE system and was considered to have a high level of evidence, presenting no methodological limitations.

Another drug that was addressed in order to verify its efficacy and safety in preventing and treating PPH was tranexamic acid (TA)^52^. The authors covering this drug report that high dosages of TA can decrease blood loss and maternal morbidity among women with PPH. Due to moderate quality evidence, this drug is recommended only in cases in which oxytocin and other uterotonics are not able to stop hemorrhaging.

Another product technology that was assessed in European countries was the transparent plastic collection bag. A multicenter RCT assessed its efficacy in preventing severe PPH, based on the measurement of blood lost after vaginal birth. The results reveal no significant differences when blood loss was measured using a collector bag or only visually verified. The authors note that more studies are needed to develop strategies able to decrease severe PPH by improving care management^53^.

In regard to process technologies, we initially verified that some authors conducted RCTs^54,60^ and an observational study^61^ to verify changes in hematological parameters caused by blood loss among women who received AMTSL (Active Management of the Third Stage of Labor) and the expectant management of the third stage of labor. They concluded that blood loss was greater and the level of hemoglobin was lower in the group with expectant management.

Active management of the third stage of labor is especially indicated for primiparous women, but the findings of a systematic review^54,60^ did not find valid and relevant evidence regarding the efficacy of physiological care in the third stage of labor among women at a low risk for PPH.

Decreased blood loss has a greater impact on the health of women in low-income countries^54,60^; however, if active management is the preferred option for AMTSL among low-risk women in high-level hospitals in developed countries, the only benefit will be to decrease drops in hemoglobin caused by childbirth^54^.

Another technology used to prevent PPH is the controlled cord traction (CCT) adopted in vaginal births to superficially decrease blood loss and reduce the duration of the third stage of labor. A multicenter RCT investigated the simplified approach of AMTSL without CCT and its results show that the risk of PPH, rate of manual removal of placenta, mean blood loss, and duration of the third stage of labor were greater in the group in which CCT was not performed^56^. Not performing CCT led to an increase in the risk of severe hemorrhaging, especially when compared to the effect of oxytocin, which is the main component of AMTSL. In regard to this process technology, more clinical studies are needed to verify whether CCT decreases blood loss and prevents PPH among women who received prophylactic oxytocin in the third stage of labor^56^.

Still in regard to the prevention of PPH, the efficacy of skin-to-skin contact and breastfeeding after birth in decreasing PPH rates were also investigated. Risk for PPH decreased by almost four times among women practicing these. The highest effect in this study was among women at a lower risk of PPH. Both practices, when implemented immediately after birth, might be efficacious in decreasing PPH rates, regardless of the already existing risk factors for PPH^62^.

According to the authors, these practices promote the release of endogenous oxytocin, and they emphasize that pregnant women should be educated and supported in the implementation of these practices during the third and fourth stage of labor^62^. Note, however, these that the application of such practices should include a rigorous assessment of the clinical conditions of the women, because such a resource is not viable for those with at-risk pregnancies.

In regard to technology intended to prevent and control severe PPH and maternal morbidity and mortality, one paper addressed here focuses on sustained trans-abdominal uterine massage. The multicenter RCT^57^ verified whether this technology can decrease blood loss after vaginal birth. The results show that patients who underwent vaginal birth, having received uterine massage combined with uterotonics, did not experience a decrease in blood loss when compared to the administration of uterotonics only. The group of women who received uterine massage reported pain and/or discomfort when receiving the massage and asked for it to be stopped^57^. Routine uterine massage is not a technology indicated for the prevention of PPH after vaginal childbirth. It is a time-consuming and painful procedure and eliminating this practice from AMTSL benefits the obstetrical team because, in addition to saving effort, the time used in its application can be directed to other tasks^57^.

Another technology addressed is an educational intervention. Whether the implementation of a protocol of early PPH prevention decreased the incidence of severe PPH was assessed. The pregnant women involved were randomly assigned to the educational intervention (where sensitization meetings were held and the protocol was discussed) or only received the protocol without interventions. The results show that the mean rate of severe PPH did not differ in the units that received the educational intervention. Some elements of the PPH prevention protocol, however, were more frequently used in the units that received the intervention, such as asking for the help of specialized personnel and asking for specialized service within 15 minutes of PPH being diagnosed^58^. The authors emphasize that educational interventions are increasingly necessary to improve clinical practices in the face of new technologies and changes implemented in care components.

Some limitations were identified in this systematic review. A total of 39 RCTs were found and, *a priori*, one assumes these present equivalent scientific rigor and evidence. After applying the GRADE system, however, a lack of methodological rigor was identified in 13 studies, which were classified as presenting low level evidence. Additionally, among the 34 papers classified as product technology, most is represented by pharmacological products.

Still, the knowledge gathered here regarding the health technologies used to prevent and control hemorrhaging in the third stage of labor contributes to the development of evidence-based instruments and protocols intended to prevent and control PPH. Moreover, studies conducted by nurses in the context of clinical practice can lead to new technological developments, whether product or process technologies, in order to meet the needs of women and to reduce avoidable deaths.

Cross-referencing with other descriptors should also be considered in future studies.

## Conclusion

Product technologies of a pharmacological nature, especially uterotonics such as misoprostol and oxytocin, the studies of which compose the body of analysis, presented high and moderate evidence on the prevention and control PPH in the third stage of labor, in addition to contributing to decreased blood loss, shorter duration of the third stage of labor, improved concentration of hematocrit/hemoglobin and a reduced need for additional uterotonics.

Among the studies addressing process technologies, the active management of the third stage of labor presented high, moderate and low evidence level, while controlled cord traction presented a high level of evidence. When product technology (oxytocin) was associated with process technology (uterine massage), the level of evidence was moderate.

Therefore, the prevention and control of hemorrhaging in the third stage of labor requires studies addressing the association of product and process technologies, considering the evidence found so far concerning the contributions of these technologies. Clinical nurses should incorporate scientific evidence, conduct new systematic reviews and develop nursing protocols to provide women with the best possible care practices.
